# Geographic and socio-economic variation in markers of indoor air pollution in Nepal: evidence from nationally-representative data

**DOI:** 10.1186/s12889-019-6512-z

**Published:** 2019-02-14

**Authors:** Saruna Ghimire, Shiva Raj Mishra, Abhishek Sharma, Adugna Siweya, Nipun Shrestha, Bipin Adhikari

**Affiliations:** 1 Agrata Health and Education (AHEAD)-Nepal, Kathmandu, Nepal; 2 Nepal Development Society, Bharatpur-10, Nepal; 3 Department of Global Health, Boston University School of Public Health, Boston, MA USA; 4 Precision Health Economics, Boston, MA USA; 5 Department of Environmental and Occupational Health, University of Nevada, Las Vegas, USA; 6 Institute for Health and Sport (IHeS), Victoria University, Melbourne, Australia; 7 Mahidol Oxford Tropical Medicine Research Unit, Faculty of Tropical Medicine, Mahidol University, Bangkok, Thailand

**Keywords:** Nepal, Socio-economic status, Fuel use, Cooking, Indoor air pollution

## Abstract

**Background:**

In low-income countries such as Nepal, indoor air pollution (IAP), generated by the indoor burning of biomass fuels, is the top-fourth risk factor driving overall morbidity and mortality. We present the first assessment of geographic and socio-economic determinants of the markers of IAP (specifically fuel types, cooking practices, and indoor smoking) in a nationally-representative sample of Nepalese households.

**Methods:**

Household level data on 11,040 households, obtained from the 2016 Nepal Demographic and Health Survey, were analyzed. Binary logistic regression analyses were conducted to assess the use of fuel types, indoor cooking practices, indoor smoking and IAP with respect to socio-economic indicators and geographic location of the household.

**Results:**

More than 80% of the households had at least one marker of IAP: 66% of the household used unclean fuel, 45% did not have a separate kitchen to cook in, and 43% had indoor smoking. In adjusted binary logistic regression, female and educational attainment of household’s head favored cleaner indoor environment, i.e., using clean fuel, cooking in a separate kitchen, not smoking indoors, and subsequently no indoor pollution. In contrast, households belonging to lower wealth quintile and rural areas did not favor a cleaner indoor environment. Households in Province 2, compared to Province 1, were particularly prone to indoor pollution due to unclean fuel use, no separate kitchen to cook in, and smoking indoors. Most of the districts had a high burden of IAP and its markers.

**Conclusions:**

Fuel choice and clean indoor practices are dependent on household socio-economic status. The geographical disparity in the distribution of markers of IAP calls for public health interventions targeting households that are poor and located in rural areas.

**Electronic supplementary material:**

The online version of this article (10.1186/s12889-019-6512-z) contains supplementary material, which is available to authorized users.

## Background

Globally, every year 4 million people die prematurely from illness attributable to indoor air pollution (IAP) [1]. IAP is generated largely by the indoor burning of biomass fuels (such as wood, crop waste, and coal), for purposes like heating, cooking, or boiling water, coupled with poor ventilation practices [2]. In 2013 alone, IAP generated from solid fuels caused 81.1 million disability-adjusted life years and nearly 2.9 million deaths [3]. In developing countries, IAP is the top-fourth risk factor driving overall morbidity and mortality [4]. The biomass smoke contains several health-damaging pollutants and chemicals such as carbon monoxide, sulphur dioxide, particulate matter (PM) 10 and 2.5 μm (PM_10_ and PM_2.5_). These pollutants are linked to diseases such as stroke, ischemic heart disease, chronic obstructive pulmonary disease, pneumonia, bronchitis, and lung cancer [1, 5].

Despite being linked to adverse health events, biomass fuel remains the predominant source of energy in low-income countries [6]. In 2012, about a third of the global population, primarily in Sub-Saharan Africa and Southern Asia, used biomass fuel as a primary source of energy and this accounted for 12% of the world energy use [6, 7]. In recent years, while a detailed assessment of IAP has become possible by monitoring pollutants at the household level, such assessments are logistically challenging and expensive for low-income countries. Consequently, several epidemiological studies assessing the impact of IAP use indirect/proxy measures, such as indoor burning of solid mass, to assess IAP [8–10].

Research on solid fuel-related pollutants is limited in Nepal [11]. A previous study, using DataRAM pDR-1000 and LASCAR-CO data logger monitoring technique, highlighted household air pollution to be a severe issue in rural areas of Nepal [12]. However, this study was limited to rural areas and southern Nepal [12]. Another study from Chitwan, Nepal, demonstrated biomass as a source of ambient endotoxins, and higher levels of endotoxin related to biomass burning were accompanied by increased levels of anti-inflammatory agents [13]. The domestic indoor air quality levels associated with biomass fuel combustion exceeds the WHO Indoor Air Quality standards and are in the hazardous range for human health [14, 15]. Given that about 80% of households in Nepal use biomass fuel [16], in a setting with limited ventilation and that the domestic PM levels in Nepalese households exceed international standards for ambient air quality [14, 15], it is crucial to shed more lights on this important issue. In fact, household air pollution from cooking with solid fuels was the third largest contributor to the burden of diseases in Nepal and was responsible for around 15,000 premature deaths in 2013 [3].

While the use of solid (unclean) fuel increases the risk of adverse health events, even using clean fuel but not in a separate kitchen space has been reported to be associated with increased IAP exposure and health risk [17–20]. A study from rural Nepal monitored the particulate matter (PM) concentrations by kitchen type (inside vs. outside of the main house) and found that 24-h average indoor PM concentrations in both kitchens types exceeded Nepal’s indoor air quality standards and the WHO PM2.5 guidelines [21]. The concentration rose steeply during the first half hour of cooking, then decreased slightly and finally levelled off to the non-cooking period concentrations [21]. The particular matter emission is higher when burning such fuels in inefficient traditional cook stoves [22]. Conversely, use of separate dwelling as a kitchen (separate kitchen) is suggested as an alternative to reduce the impact of indoor air pollution in health. Therefore, besides just the fuel types used, studies must also consider “clean cooking practice” when assessing IAP and its determining factors. Another important aspect adding to the IAP is the environmental tobacco smoke (ETS, also called second-hand smoke) [23]. The harmful effects of ETS on the non-smoking population, such as respiratory and cardiovascular disease and premature death, is well documented [24]. However, no previous studies have included ETS when measuring IAP.

Previous studies have reported that the adoption of cleaner fuels and levels of IAP are dependent on household’s socioeconomic status (SES) and geographical regions [10, 25]. In this regard, we present the first and comprehensive assessment of geographic and socio-economic determinants of the markers of IAP (specifically fuel types, cooking practice, and indoor smoking) in representative sample of Nepalese households.

## Methods

### Study area

Nepal is a developing country situated in the South-East Asia Region and consists of three ecological zones (Mountain, Hill, and Terai). Nepal is divided into 75 districts distributed across five development regions (Eastern, Central, Western, Mid-western, and Far-western). Since the promulgation of a new constitution in 2015, Nepal is undergoing federal restructuring into seven administrative provinces that are further sub-divided into local governance units (i.e., municipal, village councils) [26]. Notwithstanding these recent changes, the districts continue to serve as important administrative divisions of the country. The de-novo local governance structures requires local evidence disaggregated for districts for planning and policy considerations.

### Data source

Using the 2016 Nepal Demographic and Health Survey (NDHS), we assessed the patterns of household-level fuel use, indoor smoking, and cooking practices at provincial and district levels. We then studied the association between these markers of IAP and various socio-economic factors. The NDHS is part of the worldwide Demographic and Health Surveys Program, implemented in 90 countries, and collects information on a wide range of populations’ socio-economic and health indicators [27]. NDHS is conducted every 5 years (NDHS 2001, 2006, 2011, and 2016). The 2016 NDHS consists of a nationally-representative sample of the population (aged 15–49 years), which is also representative at the provincial levels, ecological zones, and development regions [28]. The 2016 NDHS used multi-stage stratified cluster sampling (two-stage in rural areas and three-stage in urban areas). The seven provinces of Nepal were stratified into urban and rural areas, yielding 14 sampling strata for the 2016 NDHS. In each stratum, wards - that serve as the cluster or the primary sampling units (PSU) - were selected independently by using probability proportional to size technique, yielding a total of 383 PSU. In the last stage of sampling, 30 households from each PSU were selected with an equal probability systematic selection [28]. Of the 11,490 households allocated, 11,040 were successfully interviewed [28]. Information on the variables of interest for our analyses was acquired at the household level. The 2016 NDHS data collection took place from June 19, 2016, to January 31, 2017, and the details of its methodology are documented elsewhere [28].

### Variables

Our analyses utilized household-level data on markers of IAP such as indoor smoking, fuel types used, and cooking practice, along with demographic and socioeconomic information.

#### Fuel types

The respondents were inquired about the primary fuel used for cooking in the household, to which there were 10 response categories (electricity, liquefied petroleum gas or LPG, natural gas, biogas, kerosene, charcoal, wood, straw/shrubs/grass, agricultural crop, and animal dung). For our analyses, we dichotomized these fuel types into ‘unclean fuel’ and ‘clean fuel’ as per the 2016 NDHS guidelines [28]. Clean fuel included electricity, LPG, natural gas, and biogas. A small proportion of the participants (*n* = 28, 0.4%) indicated not cooking food in the household and were thus not included in the analyses for fuel types.

#### Indoor cooking practice

Regarding cooking practices, respondents were inquired if they cooked food either in the house, or in a separate building, outdoors, or other (no food cooked in household). Only to those who responded cooking inside the house, a follow-up question inquired if the household has a separate room used as the kitchen (yes/no response). Accordingly, indoor cooking practice was dichotomised as in a separate kitchen (cooking inside the house but in a separate room used as a kitchen) and no separate kitchen (cooking inside the house but in the absence of a separate kitchen).

#### Indoor smoking

The frequency of household members smoking inside the house could be: never, daily, weekly, monthly, or less than once a month. For our analyses, we dichotomized these data into presence or absence of indoor smoking.

#### Indoor air pollution

A given household was labeled as having IAP if they had at least one of the three markers: use of unclean fuel, no separate kitchen to cook in, and presence of indoor smoking.

#### Explanatory variable

The explanatory socio-demographic variable selected for analyses include age, sex, and education level of the head of household. We used information of household head and not individuals because decisions about the fuel use and cooking practice are often made by the household head solely or in consultation with other members, and is in line with a previous study from Afghanistan [29]. Other variables include household’s wealth quintile, urban or rural locality, provinces, ecological region, developmental region, and eco-developmental region. Wealth index was calculated using the principal component analysis and based on the housing characteristics (e.g. drinking water source, toilet facilities, and flooring materials) and the number and types of consumer goods (e.g. television, bicycle, car etc.) owned by the household, [28]. Then, wealth quintiles are compiled by dividing the distribution into five equal categories, each comprising 20% of the population.

### Statistical analyses

Sample weights were adjusted according to the 2016 NDHS guidelines to generate a nationally representative sample [28]. Rao-Scott Chi-Square test was used to test the differences in fuel types, cooking practices, indoor smoking, and IAP, by explanatory variables characteristics. Univariate and multivariable logistic regressions were used to compute odds ratios (ORs) and 95% confidence interval (CI). Multivariable models were adjusted for age, sex, and education level of the house hold head and household wealth quintiles. Each of the geographical variables (provinces, developmental region, ecological region, and eco-developmental region) were evaluated in separate models. Data analyses was performed using the survey procedures that account for the weights and complex survey design of the 2016 NDHS, in SAS 9.4 (SAS Institute Inc., Cary, NC) at an alpha significance level of 0.05. The maps, showing the geographic distribution of IAP markers by districts of Nepal, was created in SAS v9.4 using gmap procedure [30]. The details of the province and districts along with their estimates and 95% CI are provided in Additional file 1: Table S1.

## Results

### Fuel types

**Fig. 1:**
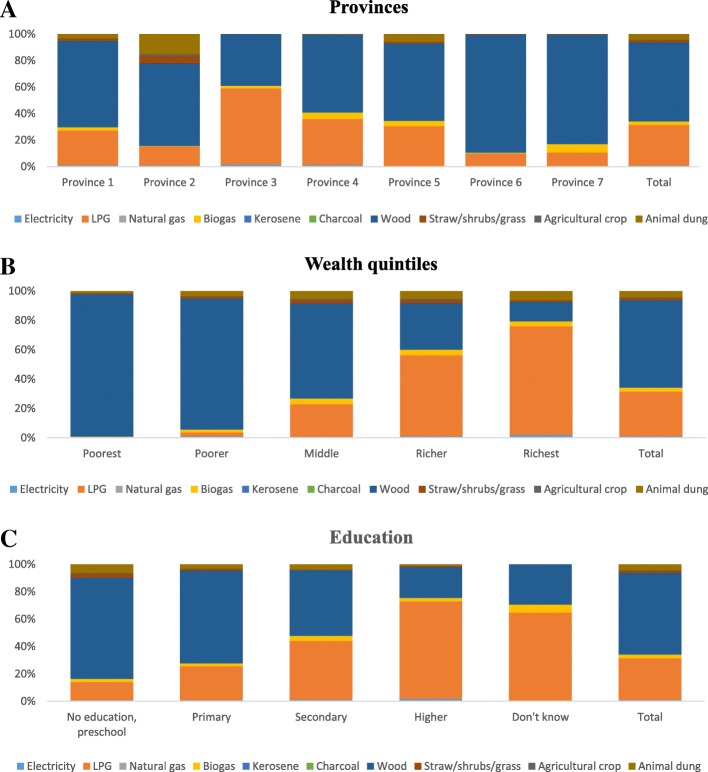
Fuel use patterns by provinces (**a**), wealth quintile (**b**), and education level (**c**) - 2016 Nepal Demographic and Health Survey (*N* = 11,012)

Nearly 60% of households relied on wood for cooking; the distribution of these was the highest in Province-1 (11.8%), among those in the lowest wealth quintile (19.5%), and among those where the household head had no education (28.9%) (Fig. 1a-c). LPG was used by about 30% of the overall households; these were mostly in Province-3 (13.1%), those that belonged to the highest wealth quintile (14.1%) and had educated household head (secondary education: 11.4% and higher education: 8.1%) (Fig. 1a-c).

**Fig. 2:**
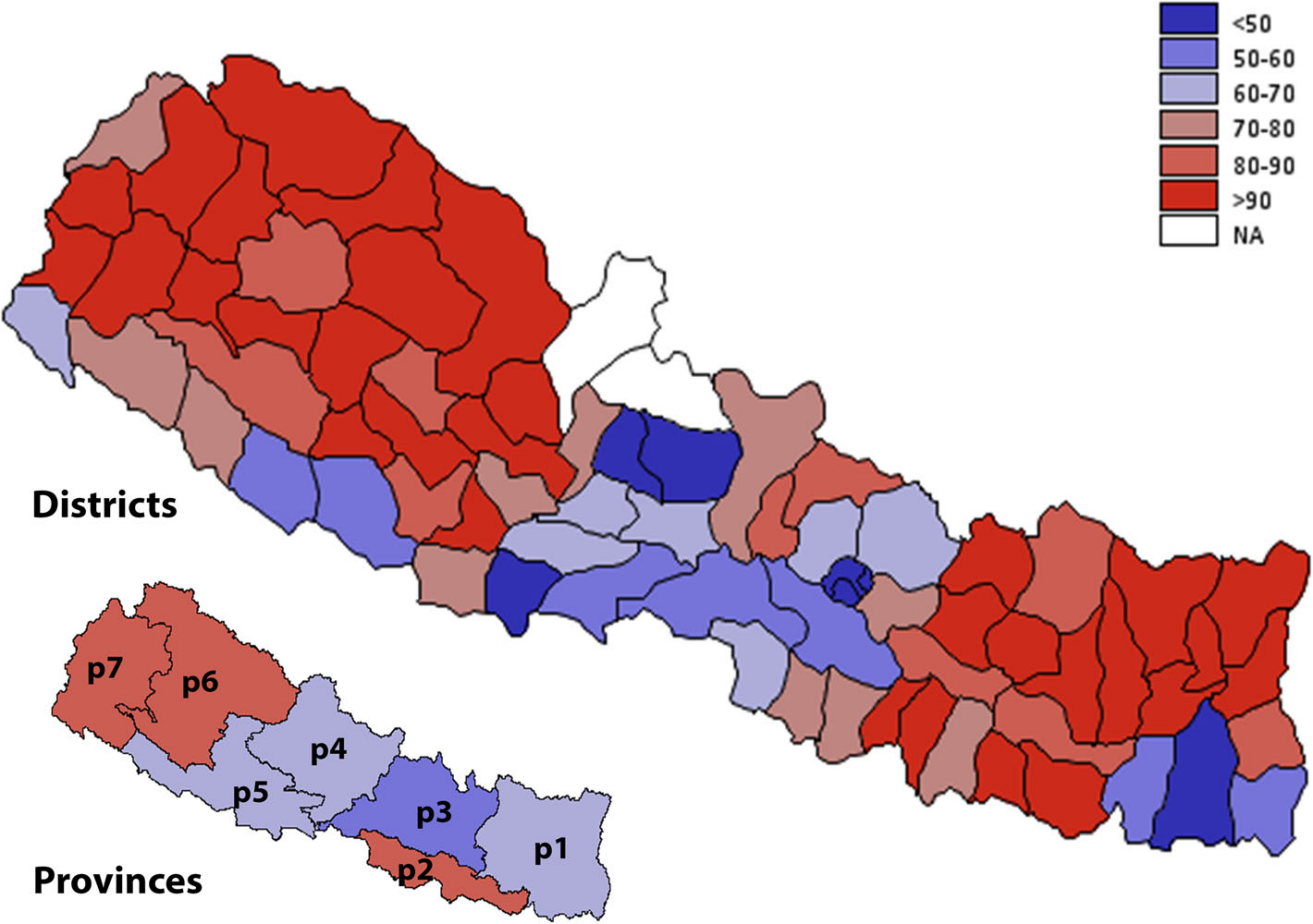
Geographical distribution of unclean fuel use by districts and provinces of Nepal. The prevalence is reported in percentage and is divided into six groups (< 50%, 50–60%, 60–70%, 70–80%, 80–90, > 90%). Estimates for two districts Manang, and Mustang were not available (NA). Refer to methods section for definition of variables, and Additional file 1: Table S1 for district and province wise estimates and their 95% CI. The map is created using GMAP procedure in SAS 9.4

Only 34% of the households used clean fuel (Table 1)*.* Table 1 shows the distribution of household fuel types by socio-demographic and geographical characteristics. In adjusted binary logistic regression, the odds of using clean fuel was higher in the households headed by a female and those having any level of formal education (Table 2). However, households headed by older age groups compared to the youngest age group (15–24 years) had lower odds of using clean fuels (Table 2). The households belonging to lower wealth quintiles and located in rural areas had lower odds of using clean fuels. Households in Province 2 had lower odds of using clean fuels but those in Province 3 and 4 had higher odds. Households from Central, Western, and Mid-western development regions were more likely to use clean fuels compared to those in the Eastern development region (Table 2). Figure 2 (A, B) shows the geographic distribution of unclean fuel use by districts and provinces. Most of the districts from Provinces 1, 2, 6 and 7 have over 80% of households using unclean fuel.

**Table 1:** Distribution of markers of indoor air pollution by key socio-demographic, geographic and ecological variables: 2016 Nepal Demographic and Health Survey

Variable	Fuel type			Cooking practice			Indoor smoking			Indoor air pollution		
	Clean (3282, 34%)	Unclean (7730, 66%)	*p*-value	Separate kitchen (4229, 55.5%)	No separate kitchen (3188, 44.5%)	*p*-value	No (5992, 56.9%)	Yes (5048, 43.1%)	*p*-value	No (1705, 17.0%)	Yes (9335, 83.0%)	*p*-value
	n (%)	n (%)		n (%)	n (%)		n (%)	n (%)		n (%)	n (%)	
Sex of household head												
Male	2175 (67.9)	5254 (69.1)	0.4118	2811 (68.2)	2097 (67.4)	0.5803	3761 (64.8)	3690 (73.8)	< 0.001	1132 (67.9)	6319 (68.8)	0.5724
Female	1107 (32.1)	2476 (30.9)		1418 (31.8)	1091 (32.6)		2231 (35.2)	1358 (26.2)		573 (32.1)	3016 (31.2)	
Age of household head in, years												
15–25	278 (8.48)	321 (4.02)	< 0.001	163 (3.7)	301 (9.7)	< 0.001	412 (7.1)	202 (3.8)	< 0.001	85 (4.9)	529 (5.8)	0.0166
25–35	787 (24.4)	1444 (18.2)		809 (18.5)	743 (24.4)		1417 (23.5)	820 (16.1)		363 (21.2)	1874 (20.1)	
35–45	781 (23.7)	1774 (23.0)		976 (23.5)	664 (20.4)		1503 (25.2)	1052 (20.5)		442 (26.0)	2113 (22.6)	
45–55	681 (20.8)	1684 (21.7)		1002 (23.9)	581 (17.7)		1150 (18.8)	1218 (24.7)		383 (22.2)	1985 (21.2)	
55–65	448 (12.8)	1392 (18.3)		721 (16.7)	501 (15.5)		830 (13.9)	1010 (19.7)		244 (14.1)	1596 (16.9)	
> 65	307 (9.85)	1115 (14.8)		558 (13.6)	398 (12.3)		680 (11.5)	746 (15.2)		188 (11.7)	1238 (13.4)	
Education level of household head												
No education, preschool	642 (18.6)	3775 (49.6)	< 0.001	1439 (31.4)	1484 (45.5)	< 0.001	2078 (33.8)	2345 (46.0)	< 0.001	289 (15.9)	4134 (43.8)	< 0.001
Primary (Grade 1–5)	585 (18.3)	1976 (24.9)		950 (22.1)	756 (22.4)		1167 (19.4)	1394 (26.7)		238 (14.8)	2323 (24.2)	
Secondary (Grade 5–10)	1231 (37.4)	1608 (21.2)		1254 (31.5)	646 (21.7)		1798 (30.3)	1048 (21.9)		668 (39.0)	2178 (24.2)	
Higher (Above grade 10)	818 (25.3)	366 (4.27)		583 (14.8)	300 (10.2)		948 (16.4)	251 (5.1)		509 (30.2)	690 (7.7)	
Don’t know	6 (0.36)	5 (0.08)		3 (0.2)	2 (0.2)		1 (0.0)	10 (0.4)		1 (0.1)	10 (0.2)	
Wealth quintile												
Quintile 1	19 (0.47)	2744 (30.2)	< 0.001	943 (17.1)	1290 (32.3)	< 0.001	1002 (13.2)	1764 (29.1)	< 0.001	4 (0.2)	2762 (24.1)	< 0.001
Quintile 2	137 (3.26)	2289 (29.0)		926 (19.5)	630 (18.9)		1152 (17.4)	1277 (24.0)		39 (1.9)	2390 (24.0)	
Quintile 3	616 (15.7)	1579 (22.3)		675 (15.3)	511 (17.7)		273 (20.8)	927 (19.2)		220 (11.0)	1980 (21.9)	
Quintile 4	1147 (36.3)	777 (12.5)		681 (18.8)	576 (23.6)		1303 (24.5)	637 (15.6)		505 (28.5)	1435 (19.1)	
Quintile 5	1363 (44.3)	341 (5.94)		1004 (29.3)	181 (7.5)		1262 (24.1)	443 (12.1)		937 (58.4)	768 (10.9)	
Residency												
Urban	2824 (86.3)	4129 (48.5)	< 0.001	2867 (68.3)	1824 (57.0)	< 0.001	3985 (64.3)	2993 (57.7)	0.0033	1458 (85.1)	5520 (56.6)	< 0.001
Rural	458 (13.7)	3601 (51.5)		1362 (31.7)	1364 (43.0)		2007 (35.7)	2055 (42.3)		247 (14.9)	3815 (43.4)	
Provinces												
Province 1	530 (15.7)	1140 (19.4)	< 0.001	637 (17.8)	353 (12.9)	< 0.001	1001 (18.8)	674 (17.3)	< 0.001	335 (19.6)	1340 (17.9)	< 0.001
Province 2	285 (8.36)	1336 (23.3)		270 (7.9)	401 (16.5)		998 (20.0)	628 (16.0)		174 (10.0)	1452 (19.9)	
Province 3	819 (40.9)	818 (13.5)		706 (26.6)	645 (30.2)		828 (21.8)	811 (24.2)		343 (32.5)	1296 (20.9)	
Province 4	561 (12.7)	930 (9.52)		695 (13.3)	417 (9.8)		906 (11.4)	592 (9.7)		300 (13.7)	1198 (10.0)	
Province 5	577 (16.5)	1050 (16.1)		736 (19.2)	490 (16.5)		1040 (18.3)	591 (13.5)		324 (18.6)	1307 (15.8)	
Province 6	240 (1.70)	1245 (7.62)		630 (6.7)	472 (6.2)		572 (3.4)	916 (8.6)		110 (1.6)	1378 (6.4)	
Province 7	270 (4.16)	1211 (10.4)		555 (8.5)	410 (7.9)		647 (6.4)	836 (10.8)		119 (4.0)	1364 (9.2)	
Ecological region												
Mountain	114 (2.80)	797 (9.31)	0.0012	436 (8.7)	335 (8.6)	0.6546	303 (4.3)	608 (10.7)	< 0.001	43 (2.1)	868 (8.1)	0.0005
Hill	1550 (53.1)	3745 (43.1)		2340 (55.7)	1756 (53.4)		2740 (44.2)	2571 (49.5)		723 (45.7)	4588 (46.7)	
Terai	1618 (44.1)	3188 (47.6)		1453 (35.7)	1097 (38.0)		2949 (51.4)	1869 (39.8)		939 (52.2)	3879 (45.2)	
Developmental region												
Eastern	565 (16.8)	1578 (26.9)	< 0.001	654 (18.3)	400 (14.9)	0.0004	1249 (23.6)	899 (23.2)	< 0.001	354 (20.7)	1794 (24.0)	< 0.001
Central	1069 (48.2)	1716 (29.3)		959 (34.0)	999 (44.8)		1578 (37.0)	1214 (34.2)		498 (41.4)	2294 (34.6)	
Western	936 (23.8)	1487 (18.5)		1109 (24.5)	714 (20.2)		1570 (23.5)	864 (16.2)		526 (27.1)	1908 (19.0)	
Mid-western	442 (7.05)	1738 (14.8)		952 (14.7)	665 (12.3)		948 (9.6)	1235 (15.5)		208 (6.8)	1975 (13.2)	
Far-western	270 (4.16)	1211 (10.4)		555 (8.5)	410 (7.9)		647 (6.4)	836 (10.8)		119 (4.0)	1364 (9.2)	
Eco-developmental region												
Eastern mountain	11 (0.48)	137 (2.56)	< 0.001	76 (2.5)	38 (1.5)	0.0002	62 (1.4)	86 (2.5)	< 0.001	5 (0.5)	143 (2.1)	< 0.001
Central mountain	55 (1.59)	168 (2.39)		77 (1.9)	100 (3.3)		74 (1.3)	149 (3.2)		19 (1.1)	204 (2.3)	
Western mountain	48 (0.72)	492 (4.36)		283 (4.3)	197 (3.7)		167 (1.7)	373 (5.0)		19 (0.5)	521 (3.6)	
Eastern hill	30 (1.11)	508 (9.05)		193 (5.9)	174 (6.6)		252 (5.2)	286 (7.8)		19 (1.4)	519 (7.3)	
Central hill	699 (36.6)	568 (9.31)		575 (22.7)	504 (24.6)		676 (18.5)	593 (18.8)		285 (28.1)	984 (16.7)	
Western hill	577 (13.3)	1053 (12.2)		806 (16.7)	461 (11.8)		1009 (13.7)	632 (11.2)		305 (14.2)	1336 (12.3)	
Mid-western hill	233 (1.88)	1176 (8.49)		583 (7.5)	437 (6.7)		616 (4.8)	796 (8.1)		108 (1.9)	1304 (7.1)	
Far-western hill	11 (0.15)	440 (4.04)		183 (2.9)	180 (3.7)		187 (2.0)	264 (3.7)		6 (0.2)	445 (3.2)	
Eastern terai	524 (15.2)	933 (15.3)		385 (9.9)	188 (6.7)		935 (17.0)	527 (13.0)		330 (18.8)	1132 (14.6)	
Central terai	315 (10.0)	980 (17.6)		307 (9.5)	395 (16.9)		828 (17.2)	472 (12.2)		194 (12.3)	1106 (15.6)	
Western terai	359 (10.5)	434 (6.31)		303 (7.8)	253 (8.4)		561 (9.7)	232 (5.0)		221 (12.9)	572 (6.6)	
Mid-western terai	192 (5.01)	326 (4.34)		226 (5.2)	135 (4.0)		281 (4.3)	237 (4.9)		91 (4.7)	427 (4.5)	
Far-western terai	228 (3.44)	515 (4.00)		232 (3.3)	126 (2.1)		344 (3.1)	401 (4.7)		103 (3.5)	642 (3.9)

**Table 2:** Univariate and multivariable analyses of markers of indoor air pollution by selected sociodemographic, geographic and ecology variables: 2016 Nepal Demographic and Health Survey (N = 11,012)

Variable	Clean Fuel Use (*n* = 3282, 34%)		Cooking in separate kitchen (*n* = 4229, 55.5%)		No Indoor Smoking (*n* = 5992, 56.9%)		No Indoor Air Pollution (*n* = 1705, 17.0%)	
	Unadjusted Odds Ratio (95% CI)	Adjusted Odds Ratio (95% CI)	Unadjusted Odds Ratio (95% CI)	Adjusted Odds Ratio (95% CI)	Unadjusted Odds Ratio (95% CI)	Adjusted Odds Ratio (95% CI)	Unadjusted Odds Ratio (95% CI)	Adjusted Odds Ratio (95% CI)
^a^ Sex of house hold head								
Male	Reference		Reference		Reference		Reference	
Female	1.06 (0.93, 1.20)	**1.85 (1.52, 2.26)**	0.96 (0.85, 1.10)	**1.54 (1.32, 1.79)**	1.53 (1.38, 1.68)	**1.64 (1.47, 1.84)**	1.04 (0.90, 1.21)	**2.18 (1.79, 2.65)**
^b^ Age of household head								
15–24	Reference		Reference		Reference		Reference	
25–34	**0.64 (0.47, 0.85)**	**0.58 (0.42, 0.81)**	1.97 (1.45, 2.67)	**2.26 (1.65, 3.07)**	0.78 (0.59, 1.03)	0.93 (0.71, 1.23)	1.25 (0.94, 1.67)	**1.59 (1.07, 2.37)**
35–44	**0.49 (0.36, 0.66)**	**0.44 (0.31, 0.62)**	2.98 (2.16, 4.11)	**3.78 (2.74, 5.21)**	0.66 (0.50, 0.87)	0.88 (0.67, 1.15)	1.37 (1.02, 1.84)	**2.12 (1.44, 3.12)**
45–54	**0.45 (0.33, 0.62)**	**0.45 (0.32, 0.63)**	3.51 (2.57, 4.79)	**5.03 (3.68, 6.87)**	0.41 (0.31, 0.54)	**0.57 (0.42, 0.77)**	1.25 (0.91, 1.70)	**2.10 (1.33, 3.32)**
55–64	**0.33 (0.24, 0.46)**	**0.42 (0.29, 0.62)**	2.79 (1.99, 3.91)	**4.85 (3.43, 6.86)**	0.38 (0.28, 0.50)	**0.61 (0.45, 0.82)**	0.99 (0.69, 1.44)	**2.37 (1.48, 3.80)**
> =65	**0.32 (0.22, 0.45)**	**0.55 (0.36, 0.83)**	2.87 (2.02, 4.08)	**5.91 (4.10, 8.52)**	0.40 (0.30, 0.54)	**0.70 (0.52, 0.95)**	1.04 (0.71, 1.54)	**3.50 (2.13, 5.76)**
^c^ Education level of household head								
No education, preschool	Reference		Reference		Reference		Reference	
Primary (Grade 1–5)	**1.96 (1.69, 2.28)**	**2.08 (1.69, 2.55)**	1.43 (1.23, 1.67)	**1.81 (1.51, 2.17)**	0.99 (0.88, 1.12)	0.96 (0.85, 1.09)	1.68 (1.39, 2.03)	**1.80 (1.42, 2.28)**
Secondary (Grade 5–10)	**4.71 (4.06, 5.46)**	**3.01 (2.35, 3.86)**	2.10 (1.81, 2.45)	**2.54 (2.11, 3.05)**	1.88 (1.65, 2.15)	**1.45 (1.24, 1.69)**	4.43 (3.61, 5.44)	**3.30 (2.52, 4.32)**
Higher (Above grade 10)	**15.77 (12.31, 20.19)**	**6.61 (4.75, 9.18)**	2.09 (1.59, 2.76)	**2.39 (1.84, 3.11)**	4.43 (3.67, 5.35)	**2.87 (2.32, 3.54)**	10.83 (8.45, 13.88)	**6.13 (4.64, 8.11)**
^d^ Wealth								
Quintile 5	Reference		Reference		Reference		Reference	
Quintile 4	**0.39 (0.30, 0.50)**	**0.42 (0.33, 0.54)**	0.20 (0.15, 0.28)	**0.26 (0.18, 0.36)**	0.78 (0.67, 0.92)	**0.80 (0.69, 0.93)**	0.28 (0.22, 0.35)	**0.33 (0.26, 0.42)**
Quintile 3	**0.09 (0.07, 0.13)**	**0.11 (0.08, 0.15)**	0.22 (0.17, 0.29)	**0.29 (0.21, 0.39)**	0.54 (0.44, 0.67)	**0.61 (0.50, 0.74)**	0.09 (0.07, 0.12)	**0.12 (0.09, 0.15)**
Quintile 2	**0.02 (0.01, 0.02)**	**0.02 (0.01, 0.03)**	0.26 (0.20, 0.35)	**0.34 (0.25, 0.48)**	0.36 (0.29, 0.45)	**0.44 (0.35, 0.55)**	0.02 (0.01, 0.02)	**0.02 (0.01, 0.03)**
Quintile 1	0.00 (0.00, 0.00)	0.00 (0.00, 0.01)	0.13 (0.10, 0.18)	**0.19 (0.14, 0.26)**	0.23 (0.18, 0.28)	**0.28 (0.22, 0.35)**	0.00 (0.00, 0.00)	**0.00 (0.00, 0.01)**
^e^ Residency								
Urban	Reference		Reference		Reference		Reference	
Rural	**0.15 (0.10, 0.22)**	**0.03 (0.02, 0.05)**	**0.62 (0.49, 0.77)**	**0.67 (0.53, 0.84)**	**0.76 (0.63, 0.91)**	0.88 (0.74, 1.05)	**0.23 (0.16, 0.33)**	**0.20 (0.15, 0.27)**
^e^ Provinces								
Province 1	Reference		Reference		Reference		Reference	
Province 2	**0.44 (0.27, 0.73)**	**0.26 (0.15, 0.44)**	0.35 (0.24, 0.51)	**0.27 (0.18, 0.41)**	1.14 (0.83, 1.58)	1.12 (0.81, 1.53)	0.46 (0.28, 0.74)	**0.35 (0.22, 0.53)**
Province 3	**3.74 (2.29, 6.09)**	**5.20 (3.22, 8.42)**	0.64 (0.45, 0.91)	**0.49 (0.34, 0.72)**	0.83 (0.61, 1.11)	**0.63 (0.49, 0.82)**	1.42 (0.95, 2.14)	0.86 (0.61, 1.22)
Province 4	**1.65 (1.02, 2.66)**	**2.21 (1.37, 3.58)**	0.99 (0.70, 1.40)	0.85 (0.59, 1.22)	1.08 (0.81, 1.43)	1.03 (0.78, 1.36)	1.25 (0.83, 1.86)	1.22 (0.85, 1.76)
Province 5	1.26 (0.76, 2.09)	1.19 (0.69, 2.05)	0.85 (0.58, 1.24)	0.75 (0.48, 1.17)	1.24 (0.93, 1.66)	1.19 (0.92, 1.53)	1.08 (0.68, 1.71)	0.92 (0.63, 1.35)
Province 6	**0.28 (0.16, 0.48)**	1.37 (0.81, 2.34)	0.78 (0.53, 1.16)	1.43 (0.91, 2.22)	0.36 (0.25, 0.51)	**0.46 (0.33, 0.65)**	0.22 (0.13, 0.38)	0.94 (0.62, 1.41)
Province 7	**0.49 (0.27, 0.91)**	0.78 (0.44, 1.41)	0.79 (0.54, 1.14)	1.12 (0.77, 1.65)	0.54 (0.39, 0.75)	**0.59 (0.43, 0.79)**	0.40 (0.20, 0.82)	0.61 (0.34, 1.08)
^e^ Developmental region								
Eastern	Reference		Reference		Reference		Reference	
Central	**2.64 (1.70, 4.11)**	**2.62 (1.59, 4.30)**	0.62 (0.45, 0.85)	**0.48 (0.33, 0.68)**	1.06 (0.83, 1.36)	0.90 (0.71, 1.14)	1.39 (0.95, 2.04)	0.99 (0.69, 1.42)
Western	**2.07 (1.33, 3.23)**	**2.18 (1.34, 3.54)**	0.99 (0.71, 1.38)	0.78 (0.54, 1.12)	1.42 (1.12, 1.79)	**1.31 (1.05, 1.63)**	1.66 (1.11, 2.47)	1.41 (0.98, 2.03)
Mid-western	0.77 (0.46, 1.27)	**2.47 (1.37, 4.43)**	0.97 (0.68, 1.38)	1.45 (0.94, 2.23)	0.61 (0.47, 0.78)	**0.73 (0.57, 0.94)**	0.59 (0.37, 0.96)	1.46 (0.92, 2.32)
Far-western	0.64 (0.35, 1.16)	1.19 (0.67, 2.12)	0.88 (0.61, 1.27)	1.26 (0.87, 1.84)	0.58 (0.43, 0.78)	**0.64 (0.48, 0.84)**	0.51 (0.25, 1.03)	0.85 (0.47, 1.53)
^e^ Ecological region								
Mountain	Reference		Reference		Reference		Reference	
Hill	**4.11 (1.47, 11.49)**	2.17 (0.86, 5.47)	1.03 (0.70, 1.50)	0.74 (0.50, 1.10)	2.19 (1.59, 3.02)	**1.73 (1.33, 2.25)**	3.77 (1.48, 9.61)	1.44 (0.78, 2.67)
Terai	**3.08 (1.15, 8.27)**	0.61 (0.24, 1.54)	0.92 (0.62, 1.38)	**0.56 (0.36, 0.85)**	3.18 (2.33, 4.34)	**2.35 (1.79, 3.07)**	4.44 (1.77, 11.10)	1.24 (0.66, 2.31)
^e^ Eco-developmental region								
Central hill	Reference		Reference		Reference		Reference	
Eastern mountain	**0.05 (0.01, 0.20)**	**0.18 (0.04–0.75)**	1.74 (0.88, 3.42)	**4.15 (1.84, 9.37)**	**0.57 (0.40, 0.83)**	1.08 (0.78, 1.50)	0.13 (0.03, 0.54)	1.12 (0.33, 3.77)
Central mountain	**0.17 (0.03, 0.91)**	0.37 (0.09–1.49)	0.61 (0.30, 1.26)	0.92 (0.51, 1.64)	**0.39 (0.18, 0.86)**	**0.57 (0.33, 0.96)**	0.29 (0.07, 1.18)	0.88 (0.46, 1.68)
Western mountain	**0.04 (0.01, 0.18)**	**0.09 (0.02–0.43)**	1.27 (0.77, 2.10)	**3.36 (2.02, 5.59)**	**0.35 (0.23, 0.52)**	**0.58 (0.40, 0.82)**	0.09 (0.02, 0.37)	0.42 (0.13, 1.40)
Eastern hill	**0.03 (0.01, 0.08)**	**0.08 (0.04–0.19)**	0.96 (0.58, 1.60)	**2.09 (1.22, 3.58)**	0.68 (0.41, 1.12)	1.24 (0.76, 2.02)	0.11 (0.05, 0.29)	0.82 (0.35, 1.90)
Western hill	**0.28 (0.17, 0.46)**	**0.29 (0.18–0.46)**	**1.54 (1.10, 2.15)**	**1.95 (1.42, 2.69)**	1.25 (0.94, 1.66)	**1.63 (1.23, 2.16)**	0.69 (0.47, 1.01)	1.28 (0.91, 1.80)
Mid-western hill	**0.06 (0.03, 0.10)**	**0.19 (0.11–0.32)**	1.23 (0.84, 1.79)	**3.31 (2.26, 4.84)**	**0.60 (0.42, 0.85)**	1.06 (0.76, 1.47)	0.15 (0.08, 0.28)	1.13 (0.67, 1.91)
Far-western hill	**0.01 (0.00, 0.03)**	**0.03 (0.01–0.07)**	0.84 (0.52, 1.36)	**2.13 (1.35, 3.35)**	**0.53 (0.34, 0.83)**	1.01 (0.68, 1.51)	0.03 (0.01, 0.09)	0.28 (0.10, 0.84)
Eastern terai	**0.25 (0.15, 0.42)**	**0.11 (0.06–0.19)**	**1.62 (1.07, 2.44)**	1.45 (0.92, 2.28)	1.33 (0.99, 1.79)	**1.53 (1.15, 2.02)**	0.77 (0.50, .17)	0.87 (0.59, 1.30)
Central terai	**0.14 (0.08, 0.26)**	**0.08 (0.04–0.14)**	**0.61 (0.42, 0.89)**	**0.66 (0.45, 0.98)**	1.43 (0.99, 2.06)	**1.91 (1.36, 2.70)**	0.47 (0.27, 0.79)	0.68 (0.45, 1.04)
Western terai	**0.42 (0.22, 0.82)**	**0.19 (0.09–0.38)**	1.01 (0.59, 1.71)	0.84 (0.49, 1.44)	**1.95 (1.39, 2.74)**	**2.27 (1.71, 3.02)**	1.15 (0.67, 1.97)	1.19 (0.75, 1.89)
Mid-western terai	**0.29 (0.15, 0.56)**	**0.36 (0.16–0.84)**	1.40 (0.77, 2.53)	2.00 (0.94, 4.25)	0.90 (0.63, 1.30)	1.21 (0.88, 1.66)	0.62 (0.37, 1.06)	1.33 (0.76, 2.32)
Far-western terai	**0.22 (0.12, 0.42)**	**0.19 (0.09–0.36)**	1.74 (1.20, 2.52)	2.97 (1.95, 4.52)	0.68 (0.44, 1.07)	0.83 (0.55, 1.25)	0.53 (0.26, 1.11)	0.99 (0.53, 1.85)

NA: Could not be estimated due to low cell frequency for that category

Significant odds ratio are bolded

^a^Multivariable model was adjusted for age, education level of the house hold head and household wealth quintiles

^b^Multivariable model was adjusted for sex, education level of the house hold head and household wealth quintiles

^c^Multivariable model was adjusted for age, sex, and household wealth quintiles

^d^Multivariable model was adjusted for age, sex, and education level of the house hold head

^e^Multivariable model was adjusted for age, sex, education level of the house hold head and household wealth quintiles

### Indoor cooking practice

**Fig. 3:**
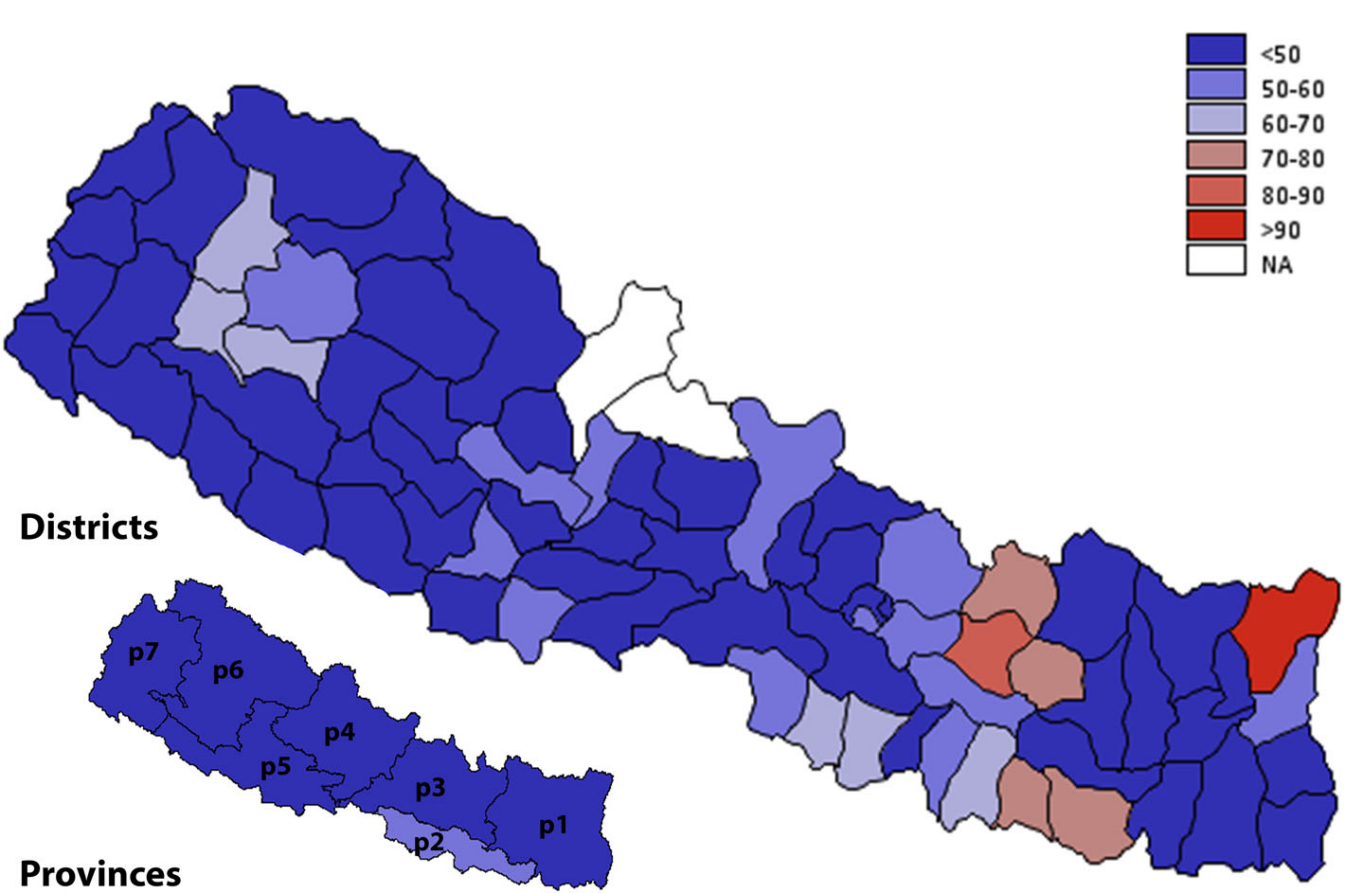
Geographical distribution of households lacking a separate kitchen for cooking by provinces and districts of Nepal. The prevalence is reported in percentage and is divided into six groups (< 50%, 50–60%, 60–70%, 70–80%, 80–90, > 90%). Estimates for two districts Manang, and Mustang were not available (NA). Refer to methods section for definition of variables, and Additional file 1: Table S1 for district and province wise estimates and their 95% CI. The map is created using GMAP procedure in SAS 9.4

About 45% of the households did not have a separate kitchen to cook in*.* Table 1 shows the distribution of household indoor cooking practice by socio-demographic and geographical characteristics. In adjusted binary logistic regression, the odds of cooking in a separate kitchen was higher in households that were headed by a female, with any level of formal education, and older age groups (Table 2). In contrast, cooking in a separate kitchen was less likely to be found in a household that belonged to lower wealth quintiles, located in rural areas, Province 2 and 3, and in the Terai region. Households in the Central development region were less likely to cook in a separate kitchen compared to those in the Eastern region (Table 2)*.* Figure 3 shows the geographic distribution of indoor cooking practice. Nearly all districts, apart from a few in Province 1, had below 50% of households which had a separate kitchen to cook in.

### Indoor smoking

**Fig. 4:**
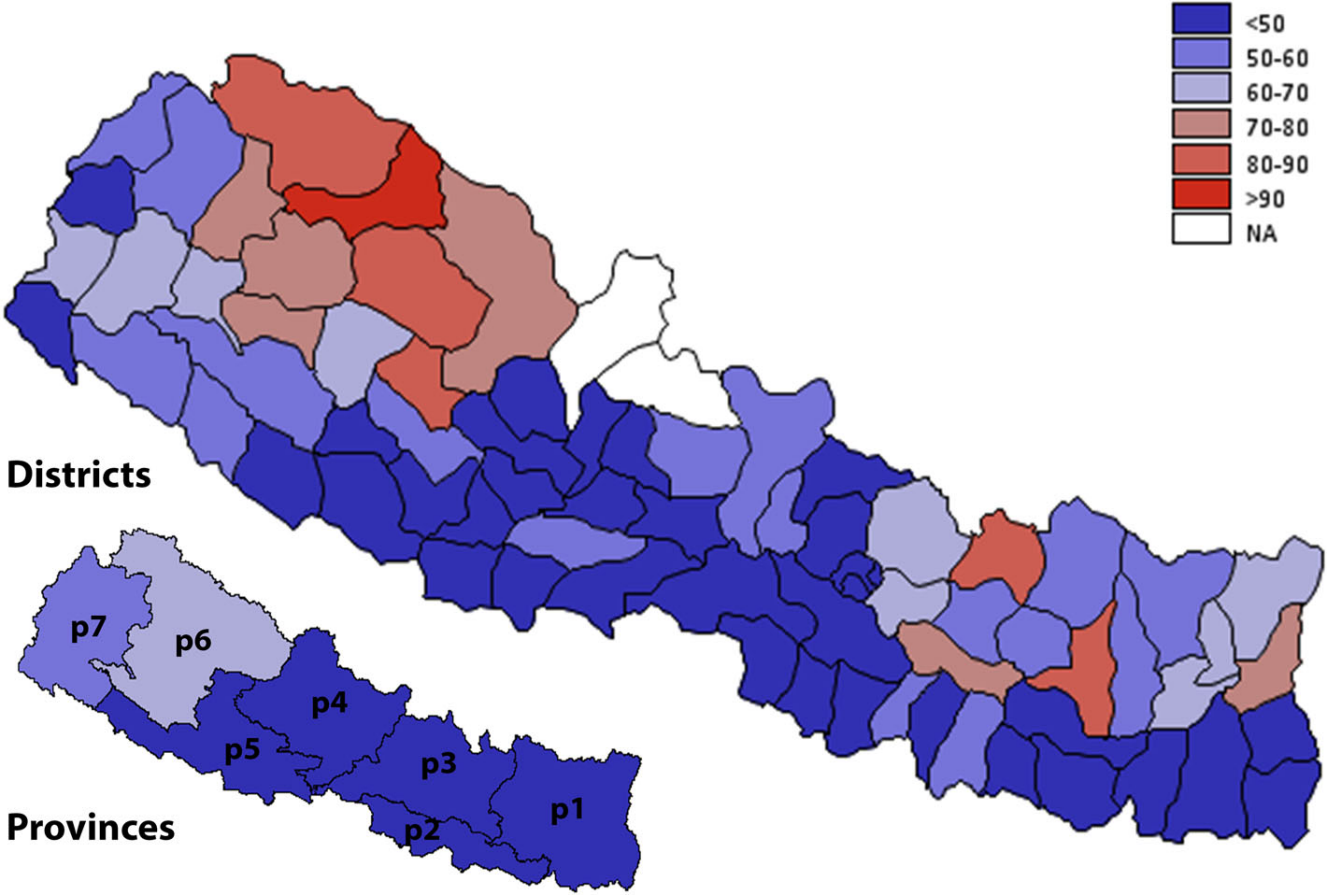
Geographical distribution of indoor smoking by provinces and districts of Nepal. The prevalence is reported in percentage and is divided into six groups (< 50%, 50–60%, 60–70%, 70–80%, 80–90, > 90%). Estimates for two districts Manang, and Mustang were not available (NA). Refer to methods section for definition of variables, and Additional file 1: Table S1 for district and province wise estimates and their 95% CI. The map is created using GMAP procedure in SAS 9.4

Overall, indoor smoking was observed in 43% of the households. Table 1 shows the distribution of indoor smoking by socio-demographic and geographical characteristics. In adjusted binary logistic regression, the odds of ‘not smoking indoor’ was higher in a household headed by a female and by members with secondary or higher education compared to those without education (Table 2). Household headed by older age groups, compared to 15–24 years, had lower odds of not smoking indoors. Likewise, a household with lower wealth quintiles compared to the highest wealth quintiles, and in Province 3, 6 and 7 compared to Province 1 had lower odds of not smoking indoors. Households in Mid- and Far-western development regions were less likely to smoke indoors compared to the Eastern region (Table 2)*.* Figure 4 shows indoor smoking by districts and provinces. In the majority of districts, the prevalence of indoor smoking was below 50%, except a few districts in Province 6 where indoor smoking was prevalent in over 70% households.

### Indoor air pollution

**Fig. 5:**
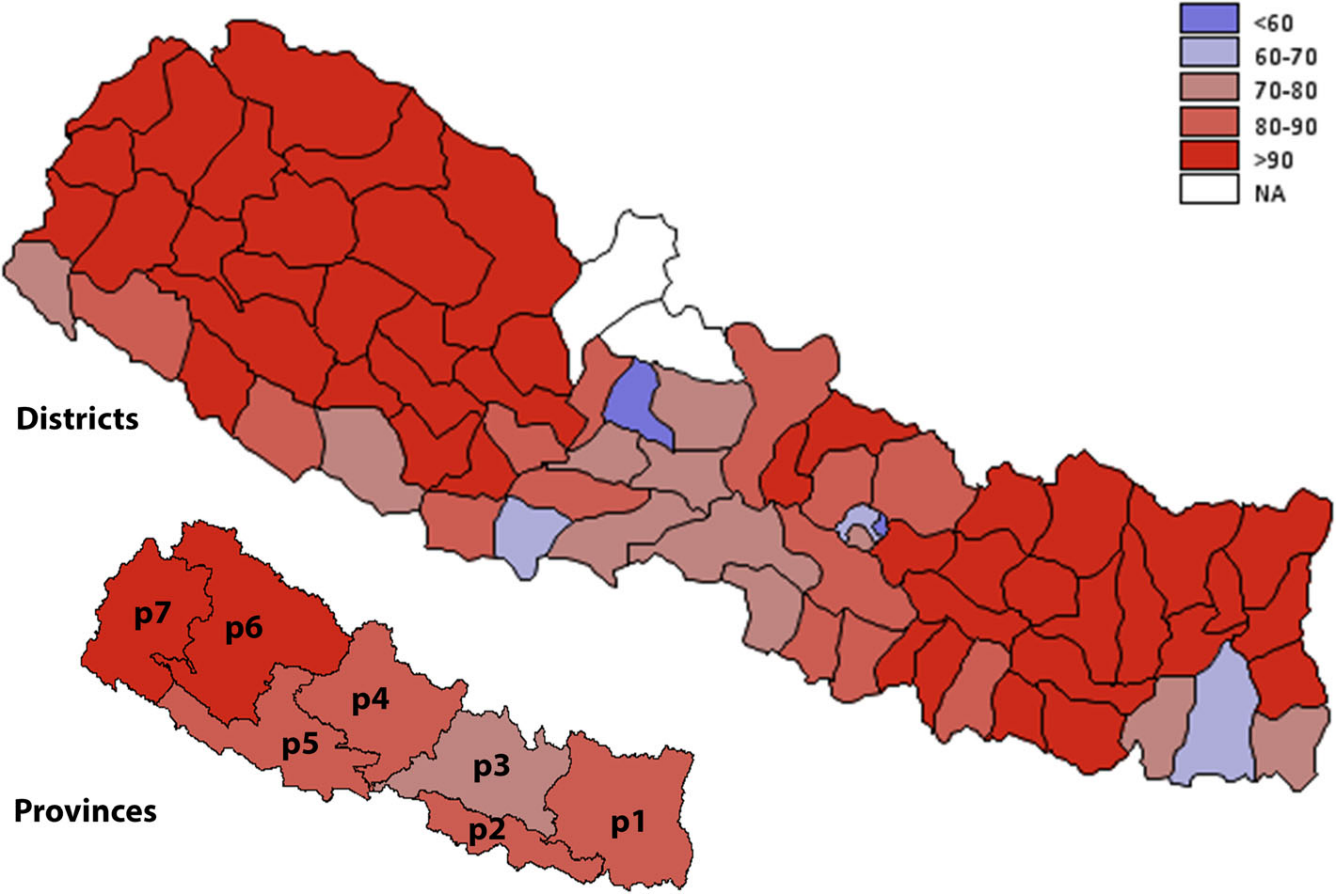
Geographical distribution of households having at least one markers of indoor air pollution (G, H) by provinces and districts of Nepal. The prevalence is reported in percentage and is divided into six groups (< 50%, 50–60%, 60–70%, 70–80%, 80–90, > 90%). Estimates for two districts Manang, and Mustang were not available (NA). Refer to methods section for definition of variables, and Additional file 1: Table S1 for district and province wise estimates and their 95% CI. The map is created using GMAP procedure in SAS 9.4

IAP was found in more than 80% of the households*.* Table 1 shows the distribution of IAP by socio-demographic and geographical characteristics. In the adjusted model, households headed by females, older age members compared to youngest age group (15–24 years), and those headed by relatively educated members had higher odds of no IAP (Table 2). In contrast, households in lower wealth quintiles compared to those in the highest quintile, in rural areas compared to urban areas, and in Province 2 compared to Province 1 were more likely to have IAP (Table 2). Figure 5 shows the geographic distribution of at least one marker at the household level. In nearly all districts, IAP was prevalent in over 80% of households.

## Discussion

This study assessed the geographic distributions and socio-economic determinants of IAP and its markers, specifically, fuel types, cooking practices, and indoor smoking in Nepal. The prevalence of unclean fuel use, not having a separate kitchen to cook in, indoor smoking indicate a high IAP in Nepal. Socio-demographic characteristics of the household head influenced indoor environment; the household head being female and having a formal education favored a cleaner indoor environment. Poor household economy (as measured by wealth quintiles) and being located in rural areas was associated with an unclean indoor environment. There also exists a geographic variation in the distribution of IAP and its markers.

The findings that there exists a high burden of unclean fuel types, lack of a separate kitchen to cook in, presence of indoor smoking, and subsequent IAP in Nepal was somewhat expected and is supported by previous studies [11, 12, 31]. Notably, around 60% of the households used solid fuel. Burning biomass fuel for daily activities such as cooking and heating is common in Nepal, especially among rural households [16, 31, 32]. Nepal ranks as the 17th poorest country, with less than $1000 gross domestic income per capita. Clean fuels are expensive compared to solid fuels [33]. Therefore, the majority of Nepalese households use cheaper, solid (unclean) fuels (such as wood, dung and crop residues) which are cheaper and more widely available [34].

Our findings that fuel choice, cooking practices and indoor smoking are significantly associated with households SES are in line with a previous systematic review [25]. Illiteracy coupled with poverty seem to have a role in unclean choices made by households. The overall literacy rate in Nepal is 62%, and it is significantly lower in the rural areas [35]. Given that educated individuals might be aware of the well-established harmful health impact of IAP [1, 5], households with higher income paired with an educated household head are more likely to use expensive, cleaner, and safer fuel [25].

We also observed that clean fuel use and cooking in a separate kitchen were less common in rural areas than in urban areas. These findings are in line with previous studies [25, 36], and may be explained by the fact that rural settings have limited availability of clean fuels and access to forest wood/fuel is easier [25]. Additionally, overall poverty rates and illiteracy are higher in rural Nepal [35, 37]. Thus, poverty and illiteracy coupled with limited clean-fuel access might explain the rural-urban difference in practices contributing to clean indoor air.

In our study, households with a female head were more likely to use cleaner fuel and cook in a separate kitchen, which is in line with a previous study [25]. However, in our study, the majority of the studied households had male heads and with a prevalence of unclean choices. Nepal is a patriarchal society where the household decision-making is primarily confined to male members and females are obligated to follow the decisions [38, 39]. In a household headed by a female, the decision to choose a smokeless stove was common.

Analysis by geography suggests a variation in the distribution of IAP and its markers. Although, most of the districts and provinces individually have a high burden of IAP and its markers, households in Province 2 (referenced to Province 1) were particularly prone to indoor pollution and/or its markers whereas households in Central development region (referenced to the Eastern region) used clean fuels but lacked a separate kitchen to cook. The geographical disparity in the indoor environment is closely linked with the socio-economic disparities by the regions of the country. Recent estimates reveal that Province 2 is the poorest province in terms of poverty intensity and the percentage of the population that is in severe poverty [40]. Furthermore, there is a disharmony in the level of industrialization and urbanization by geographical regions of Nepal [41], which may lead to geographical variation in peoples’ access to clean energy options [42]. Since many of the most developed cities of Nepal lie in the central development region, access to clean fuel is higher and supply of woods as fuel is low due to deforestation and urbanization. This region also has a higher literacy rate and more employment opportunities. However, due to rapid population growth and unplanned urbanization, people, especially in the Kathmandu valley of this region, are forced to rent/own small spaces and may not have space for a separate kitchen [16]. Similarly, education is crucial for the adoption of cleaner energy options [25]. However, literacy rate also varies substantially between Nepal’s districts and regions [35], with the highest literacy rate being in the central development region. In another district, Rautahat, of Province 2, only 42% of the population are literate [16]. Further, the North to South division of the country into three altitude-based divisions, namely Mountain, Hill and Terai, shows substantial variation in geography, climate, biodiversity, and socio-cultural and lifestyle activities [43]. For example, the Mountain region, also known as the Himalayas in the west, contains eight of the world’s ten tallest mountains, including the Everest, and more than 240 peaks over 6000 m [16]. The climate is often cold, and houses are poorly ventilated. In the mountain, indoor activities are high during the cold winter whereas in Terai, indoor activities are high during summer/monsoon. Therefore, it also likely that the heterogeneity in the markers of IAP at the district and provinces level may have been brought up by the larger diversity that exists within Nepal in terms of climate, culture and behaviors that influence fuel choices, smoking and lay out of the houses [43].

### Strengths and limitations

This is a first nationally representative study exploring the extent of fuel use, practices and makers of IAP with regard to household SES and geography in the Nepalese context. Evaluating factors like indoor smoking and separate kitchen for cooking provides better estimates of IAP exposure as compared to earlier studies which were limited to fuel type households used. Geographic analyses are helpful to design targeted interventions; however, this study being cross-sectional in nature limits causal inferences. Since 2016, NDHS collected information on the primary source of fuel; there may be misclassification bias towards referring to cooking fuel and in the cases where a given household uses a combination of different fuels. The 2016 NDHS survey was not designed, primarily, for district-level analyses; there is a possibility that our geographic mapping of prevalence is underpowered for some districts where fewer households were sampled. However, we tried to overcome this limitation by providing the 95% CI of the prevalence instead of point estimate (Additional file 1) as well as by two-stage mapping, i.e. provinces and subsequently district level. The 2016 NDHS did not have two of the cooking practice variables, i.e. “Food cooked on stove or open fire” and “Household has a chimney, hood or neither.” Another important limitations is that the study defined indoor air quality based on the type of fuel used, types of kitchens, and indoor smoking practices. Future study will benefit from using more robust indicators of combustion-related air pollution such as PM samplers, and chemical analysis including black carbon, sulfate, nitrate, PAHs etc. However, they are expensive options to be used at the population-level surveys; furthermore, it requires a trained staff to operate. Therefore our methodology to charaterize household level IAP appeared as a solution to this in low income settings until a low-cost measurement is available. Further, variation in indoor air quality has been noted by geography, season, temperature, time of the day needs to be considered [14]. Therefore, future studies need to be location specific as well as consider multiple measurements to capture aforementioned variability.

Despite the limitations, our study provides new insights into socio-economic and geographic determinants of indoor air pollution in Nepal. In 2015, Nepal underwent federal restructuring and was divided into 7 provinces that serve as the administrative units. Province level data, although crucial for the de-novo Provincial government for planning and policy considerations, is limited. Our findings at the district and province level may assist policy makers, federal public health agencies, district health offices, and other stakeholders in informed decision making, lobbying, and resource prioritization. Given that data aggregated at the province level masked the heterogeneity within the districts, provincial government and future research should be cautious when making decisions based on aggregated data. For example, in Province 1, the prevalence of solid fuel use was 60–70% but many of the district in the province (namely Taplejung, Sankhuwasabha, Solukhumbu) had > 90% of solid fuel use.

## Conclusions

More than 80% of the Nepalese households had at least one markers of IAP. With the objective to explore how households’ SES and geographical location influence fuel choice and clean indoor practices, this study found that fuel choice and cooking practices are dependent on household SES; lower SES household were more likely to have IAP markers and unclean indoor air. These findings call for public health interventions targeting low SES households to reduce the burden of IAP and subsequent health events. We also note the prevailing geographical disparity in distribution of fuel choice, cooking practices and markers of IAP. The findings related to the geographical distribution of markers of IAP are first to be recorded in the Nepalese context and may help the newly established provincial government and local stakeholders to identify the needs at a local level.

## Additional file

Additional file 1: **Table S1.** District-wise distribution of fuel types, indoor cooking practice, indoor smoking, and indoor air pollution: 2016 Nepal Demographic and Health Survey (*N* = 11,012). This file provides the district wise burden estimates used in Figs. 2, 3, 4 and 5. (DOCX 58 kb)

## Abbreviations

CI: Confidence Interval
ETS: Environmental Tobacco Smoke
IAP: Indoor Air Pollution
LPG: Liquefied Petroleum Gas
NDHS: Nepal Demographic and Health Survey
ORs: Odds Ratios
PSU: Primary Sampling Units
SES: Socioeconomic Status
