# Supplementary material for: Geographic and socio-economic variation in markers of indoor air pollution in Nepal: evidence from nationally-representative data
Source: BMC Public Health. 2019 Feb 14;19:195. doi: 10.1186/s12889-019-6512-z (PMC6376789; doi:10.1186/s12889-019-6512-z)
Supplement: Supplementary file 1 — Table S1. District-wise distribution of fuel types, indoor cooking practice, indoor smoking, and indoor air pollution: 2016 Nepal Demographic and Health Survey (N = 11,012). This file provides the district wise burden estimates used in Figs. 2, 3, 4 and 5. (DOCX 58 kb) [file 12889_2019_6512_MOESM1_ESM.docx]

**TableS 1: District-wise distribution of fuel types, indoor cooking practice, indoor smoking, and indoor air pollution: 2016 Nepal Demographic and Health Survey (N=11,012).**

| **Districts** | **Clean-fuel (N=3282, 34%)** | | **Unclean-fuel (N=7730, 66%)** | | **Cooking in separate kitchen (N=4229, 55.5%)** | | **No separate kitchen to cook (n=3188, 44.5%)** | | **No Passive smoking (5992, 56.9%)** | | **Passive smoking (5048, 43.1%)** | | **No indoor air pollution (1705, 17.0%)** | | **Indoor air pollution (9335, 83.0%)** | | |
| --- | --- | --- | --- | --- | --- | --- | --- | --- | --- | --- | --- | --- | --- | --- | --- | --- | --- |
|  | **N** | **% (95%CI)** | **N** | **% (95%CI)** | **N** | **% (95%CI)** | **N** | **% (95%CI)** | **N** | **% (95%CI)** | **N** | **% (95%CI)** | **N** | **% (95%CI)** | **N** | **% (95%CI)** |  |
| **Province 1** | | | | | | | | | | | | | | | | |  |
| Taplejung | 0 | 0 | 29 | 100.0 (100.0 - 100.0) | 1 | 9.1 (9.1 - 9.1) | 10 | 90.9 (90.9 - 90.9) | 9 | 31.0 (31.0 - 31.0) | 20 | 69.0 (69.0 - 69.0) | 0 | 0 | 29 | 100.0 (100.0 - 100.0) |  |
| Panchthar | 0 | 0 | 30 | 100.0 (100.0 - 100.0) | 2 | 50.0 (50.0 - 50.0) | 2 | 50.0 (50.0 - 50.0) | 8 | 26.7 (26.7 - 26.7) | 22 | 73.3 (73.3 - 73.3) | 0 | 0 | 30 | 100.0 (100.0 - 100.0) |  |
| Ilam | 17 | 15.5 (4.3 - 26.7) | 102 | 84.5 (73.3 - 95.7) | 29 | 63.9 (45.8 - 82.0) | 18 | 36.1 (18.0 - 54.2) | 82 | 68.8 (45.7 - 91.9) | 37 | 31.2 (8.1 - 54.3) | 11 | 10.0 (2.0 - 17.9) | 108 | 90.0 (82.1 - 98.0) |  |
| Jhapa | 141 | 42.2 (21.4 - 62.9) | 174 | 57.8 (37.1 - 78.6) | 111 | 76.1 (68.2 - 84.0) | 36 | 23.9 (16.0 - 31.8) | 204 | 64.9 (57.2 - 72.6) | 112 | 35.1 (27.4 - 42.8) | 90 | 27.4 (12.8 - 42.0) | 226 | 72.6 (58.0 - 87.2) |  |
| Morang | 212 | 54.5 (37.7 - 71.2) | 167 | 45.5 (28.8 - 62.3) | 154 | 68.2 (57.9 - 78.4) | 61 | 31.8 (21.6 - 42.1) | 290 | 76.7 (71.3 - 82.1) | 89 | 23.3 (17.9 - 28.7) | 137 | 34.0 (21.7 - 46.4) | 242 | 66.0 (53.6 - 78.3) |  |
| Sunsari | 136 | 45.7 (22.9 - 68.5) | 154 | 54.3 (31.5 - 77.1) | 103 | 72.2 (59.3 - 85.1) | 44 | 27.8 (14.9 - 40.7) | 193 | 64.9 (57.7 - 72.2) | 101 | 35.1 (27.8 - 42.3) | 84 | 27.9 (13.7 - 42.0) | 210 | 72.1 (58.0 - 86.3) |  |
| Dhankuta | 1 | 1.9 (0.0 - 4.2) | 58 | 98.1 (95.8 - 100.0) | 17 | 54.6 (37.0 - 72.3) | 15 | 45.4 (27.7 - 63.0) | 22 | 39.2 (16.9 - 61.5) | 37 | 60.8 (38.5 - 83.1) | 0 | 0 | 59 | 100.0 (100.0 - 100.0) |  |
| Terhathum | 0 | 0 | 30 | 100.0 (100.0 - 100.0) | 7 | 53.8 (53.8 - 53.8) | 6 | 46.2 (46.2 - 46.2) | 11 | 36.7 (36.7 - 36.7) | 19 | 63.3 (63.3 - 63.3) | 0 | 0 | 30 | 100.0 (100.0 - 100.0) |  |
| Sankhuwasabha | 1 | 1.2 (0.0 - 3.3) | 59 | 98.8 (96.7 - 100.0) | 40 | 76.7 (75.5 - 77.8) | 12 | 23.3 (22.2 - 24.5) | 24 | 40.9 (36.7 - 45.2) | 36 | 59.1 (54.8 - 63.3) | 0 | 0 | 60 | 100.0 (100.0 - 100.0) |  |
| Bhojpur | 1 | 1.1 (0.0 - 3.1) | 59 | 98.9 (96.9 - 100.0) | 34 | 59.9 (57.4 - 62.5) | 22 | 40.1 (37.5 - 42.6) | 28 | 44.3 (36.2 - 52.4) | 32 | 55.7 (47.6 - 63.8) | 0 | 0 | 60 | 100.0 (100.0 - 100.0) |  |
| Solukhumbu | 10 | 19.6 (5.9 - 33.3) | 49 | 80.4 (66.7 - 94.1) | 35 | 70.2 (62.1 - 78.2) | 16 | 29.8 (21.8 - 37.9) | 29 | 49.8 (46.5 - 53.1) | 30 | 50.2 (46.9 - 53.5) | 5 | 9.8 (2.9 - 16.7) | 54 | 90.2 (83.3 - 97.1) |  |
| Okhaldhunga | 0 | 0 | 60 | 100.0 (100.0 - 100.0) | 11 | 21.7 (10.8 - 32.5) | 40 | 78.3 (67.5 - 89.2) | 26 | 43.0 (14.8 - 71.3) | 34 | 57.0 (28.7 - 85.2) | 0 | 0 | 60 | 100.0 (100.0 - 100.0) |  |
| Khotang | 1 | 1.1 (0.0 - 2.9) | 89 | 98.9 (97.1 - 100.0) | 46 | 53.5 (45.0 - 61.9) | 40 | 46.5 (38.1 - 55.0) | 18 | 20.0 (20.0 - 20.0) | 72 | 80.0 (80.0 - 80.0) | 0 | 0 | 90 | 100.0 (100.0 - 100.0) |  |
| Udayapur | 10 | 10.9 (1.4 - 20.4) | 80 | 89.1 (79.6 - 98.6) | 47 | 60.3 (27.6 - 93.0) | 31 | 39.7 (7.0 - 72.4) | 57 | 63.7 (60.0 - 67.5) | 33 | 36.3 (32.5 - 40.0) | 8 | 8.7 (1.1 - 16.3) | 82 | 91.3 (83.7 - 98.9) |  |
| **Province 2** | | | | | | | | | | | | | | | | |  |
| Saptari | 9 | 3.6 (0.8 - 6.5) | 226 | 96.4 (93.5 - 99.2) | 8 | 20.1 (3.3 - 36.9) | 28 | 79.9 (63.1 - 96.7) | 122 | 51.6 (44.4 - 58.8) | 113 | 48.4 (41.2 - 55.6) | 6 | 2.6 (0.5 - 4.7) | 229 | 97.4 (95.3 - 99.5) |  |
| Siraha | 26 | 10.0 (2.8 - 17.2) | 212 | 90.0 (82.8 - 97.2) | 9 | 29.1 (8.6 - 49.6) | 19 | 70.9 (50.4 - 91.4) | 126 | 51.6 (39.9 - 63.3) | 112 | 48.4 (36.7 - 60.1) | 13 | 4.4 (1.5 - 7.3) | 225 | 95.6 (92.7 - 98.5) |  |
| Dhanusa | 48 | 21.0 (0.6 - 41.4) | 177 | 79.0 (58.6 - 99.4) | 15 | 31.3 (14.7 - 47.9) | 30 | 68.7 (52.1 - 85.3) | 106 | 46.6 (38.5 - 54.8) | 121 | 53.4 (45.2 - 61.5) | 27 | 11.3 (1.2 - 21.4) | 200 | 88.7 (78.6 - 98.8) |  |
| Mahotari | 12 | 5.9 (0.8 - 10.9) | 188 | 94.1 (89.1 - 99.2) | 40 | 40.2 (24.2 - 56.2) | 62 | 59.8 (43.8 - 75.8) | 140 | 74.5 (61.9 - 87.1) | 62 | 25.5 (12.9 - 38.1) | 8 | 4.1 (1.9 - 6.4) | 194 | 95.9 (93.6 - 98.1) |  |
| Sarlahi | 21 | 9.3 (0.7 - 17.9) | 174 | 90.7 (82.1 - 99.3) | 19 | 60.8 (47.8 - 73.9) | 11 | 39.2 (26.1 - 52.2) | 93 | 45.5 (32.9 - 58.2) | 102 | 54.5 (41.8 - 67.1) | 11 | 4.8 (0.0 - 10.4) | 184 | 95.2 (89.6 - 100.0) |  |
| Rautahat | 41 | 20.6 (2.4 - 38.7) | 136 | 79.4 (61.3 - 97.6) | 52 | 37.1 (16.6 - 57.5) | 80 | 62.9 (42.5 - 83.4) | 124 | 69.9 (59.6 - 80.2) | 53 | 30.1 (19.8 - 40.4) | 26 | 13.1 (2.3 - 24.0) | 151 | 86.9 (76.0 - 97.7) |  |
| Bara | 68 | 26.4 (5.1 - 47.7) | 134 | 73.6 (52.3 - 94.9) | 70 | 34.9 (24.8 - 45.0) | 101 | 65.1 (55.0 - 75.2) | 155 | 76.4 (66.9 - 86.0) | 48 | 23.6 (14.0 - 33.1) | 44 | 16.5 (2.1 - 31.0) | 159 | 83.5 (69.0 - 97.9) |  |
| Parsa | 60 | 32.1 (8.9 - 55.3) | 89 | 67.9 (44.7 - 91.1) | 57 | 43.2 (30.4 - 56.0) | 70 | 56.8 (44.0 - 69.6) | 132 | 87.9 (84.4 - 91.5) | 17 | 12.1 (8.5 - 15.6) | 39 | 20.3 (4.6 - 36.0) | 110 | 79.7 (64.0 - 95.4) |  |
| **Province 3** | | | | | | | | | | | | | | | | |  |
| Sindhuli | 16 | 18.0 (10.1 - 25.9) | 73 | 82.0 (74.1 - 89.9) | 38 | 49.8 (40.0 - 59.7) | 38 | 50.2 (40.3 - 60.0) | 23 | 26.0 (20.9 - 31.1) | 66 | 74.0 (68.9 - 79.1) | 0 | 0 | 89 | 100.0 (100.0 - 100.0) |  |
| Ramechhap | 0 | 0 | 88 | 100.0 (100.0 - 100.0) | 12 | 14.0 (2.2 - 25.8) | 72 | 86.0 (74.2 - 97.8) | 40 | 45.0 (25.7 - 64.3) | 48 | 55.0 (35.7 - 74.3) | 0 | 0 | 88 | 100.0 (100.0 - 100.0) |  |
| Dolakha | 1 | 1.9 (0.0 - 5.0) | 44 | 98.1 (95.0 - 100.0) | 10 | 23.0 (14.9 - 31.2) | 32 | 77.0 (68.8 - 85.1) | 7 | 15.3 (12.7 - 17.9) | 38 | 84.7 (82.1 - 87.3) | 0 | 0 | 45 | 100.0 (100.0 - 100.0) |  |
| Sindhupalchowk | 51 | 35.8 (0.0 - 76.3) | 98 | 64.2 (23.7 - 100.0) | 52 | 45.1 (24.5 - 65.8) | 60 | 54.9 (34.2 - 75.5) | 52 | 37.4 (16.1 - 58.6) | 97 | 62.6 (41.4 - 83.9) | 18 | 12.8 (0.0 - 28.0) | 131 | 87.2 (72.0 - 100.0) |  |
| Kavre | 33 | 26.5 (9.5 - 43.5) | 108 | 73.5 (56.5 - 90.5) | 46 | 40.8 (27.8 - 53.7) | 69 | 59.2 (46.3 - 72.2) | 47 | 34.7 (24.1 - 45.3) | 94 | 65.3 (54.7 - 75.9) | 4 | 3.6 (0.0 - 8.0) | 137 | 96.4 (92.0 - 100.0) |  |
| Lalitpur | 83 | 100.0 (100.0 - 100.0) | 0 | 0 | 43 | 43.1 (25.0 - 61.3) | 37 | 56.9 (38.7 - 75.0) | 45 | 56.5 (49.8 - 63.3) | 38 | 43.5 (36.7 - 50.2) | 23 | 21.0 (10.5 - 31.4) | 60 | 79.0 (68.6 - 89.5) |  |
| Bhaktapur | 77 | 92.3 (82.9 - 100.0) | 7 | 7.7 (0.0 - 17.1) | 54 | 71.4 (62.6 - 80.2) | 23 | 28.6 (19.8 - 37.4) | 42 | 58.1 (30.9 - 85.2) | 42 | 41.9 (14.8 - 69.1) | 33 | 44.7 (28.2 - 61.1) | 51 | 55.3 (38.9 - 71.8) |  |
| Kathmandu | 390 | 91.9 (81.2 - 100.0) | 16 | 8.1 (0.0 - 18.8) | 188 | 56.7 (47.3 - 66.1) | 153 | 43.3 (33.9 - 52.7) | 287 | 64.4 (52.6 - 76.2) | 121 | 35.6 (23.8 - 47.4) | 179 | 38.2 (27.9 - 48.6) | 229 | 61.8 (51.4 - 72.1) |  |
| Nuwakot | 37 | 33.0 (0.0 - 68.5) | 79 | 67.0 (31.5 - 100.0) | 72 | 70.0 (67.4 - 72.6) | 31 | 30.0 (27.4 - 32.6) | 61 | 52.9 (44.6 - 61.2) | 55 | 47.1 (38.8 - 55.4) | 20 | 17.8 (0.6 - 35.0) | 96 | 82.2 (65.0 - 99.4) |  |
| Rasuwa | 3 | 10.3 (10.3 - 10.3) | 26 | 89.7 (89.7 - 89.7) | 15 | 65.2 (65.2 - 65.2) | 8 | 34.8 (34.8 - 34.8) | 15 | 51.7 (51.7 - 51.7) | 14 | 48.3 (48.3 - 48.3) | 1 | 3.4 (3.4 - 3.4) | 28 | 96.6 (96.6 - 96.6) |  |
| Dhading | 21 | 18.0 (3.5 - 32.5) | 95 | 82.0 (67.5 - 96.5) | 45 | 50.5 (40.1 - 60.9) | 44 | 49.5 (39.1 - 59.9) | 47 | 40.6 (30.0 - 51.2) | 69 | 59.4 (48.8 - 70.0) | 9 | 7.7 (0.0 - 15.5) | 107 | 92.3 (84.5 - 100.0) |  |
| Makwanpur | 42 | 46.6 (12.0 - 81.2) | 102 | 53.4 (18.8 - 88.0) | 77 | 58.8 (42.4 - 75.3) | 37 | 41.2 (24.7 - 57.6) | 84 | 67.0 (51.0 - 82.9) | 60 | 33.0 (17.1 - 49.0) | 17 | 18.4 (5.9 - 30.9) | 127 | 81.6 (69.1 - 94.1) |  |
| Chitwan | 65 | 43.3 (5.9 - 80.7) | 82 | 56.7 (19.3 - 94.1) | 54 | 53.0 (34.7 - 71.4) | 41 | 47.0 (28.6 - 65.3) | 78 | 55.7 (36.7 - 74.7) | 69 | 44.3 (25.3 - 63.3) | 39 | 26.9 (0.4 - 53.5) | 108 | 73.1 (46.5 - 99.6) |  |
| **Province 4** | | | | | | | | | | | | | | | | |  |
| Gorkha | 42 | 25.6 (7.5 - 43.7) | 118 | 74.4 (56.3 - 92.5) | 52 | 45.9 (28.0 - 63.7) | 59 | 54.1 (36.3 - 72.0) | 80 | 49.2 (38.1 - 60.3) | 80 | 50.8 (39.7 - 61.9) | 24 | 14.5 (6.2 - 22.8) | 136 | 85.5 (77.2 - 93.8) |  |
| Lamjung | 62 | 62.6 (30.4 - 94.8) | 57 | 37.4 (5.2 - 69.6) | 70 | 75.9 (71.5 - 80.3) | 21 | 24.1 (19.7 - 28.5) | 47 | 40.6 (32.2 - 49.0) | 72 | 59.4 (51.0 - 67.8) | 20 | 20.7 (6.8 - 34.6) | 99 | 79.3 (65.4 - 93.2) |  |
| Tanahu | 81 | 37.6 (15.8 - 59.4) | 158 | 62.4 (40.6 - 84.2) | 105 | 70.6 (66.4 - 74.8) | 46 | 29.4 (25.2 - 33.6) | 126 | 54.1 (46.6 - 61.6) | 114 | 45.9 (38.4 - 53.4) | 44 | 20.8 (8.7 - 32.9) | 196 | 79.2 (67.1 - 91.3) |  |
| Syangja | 57 | 32.3 (7.1 - 57.5) | 118 | 67.7 (42.5 - 92.9) | 97 | 78.1 (68.6 - 87.5) | 28 | 21.9 (12.5 - 31.4) | 124 | 70.6 (60.8 - 80.4) | 51 | 29.4 (19.6 - 39.2) | 38 | 21.5 (7.0 - 36.0) | 137 | 78.5 (64.0 - 93.0) |  |
| Kaski | 239 | 76.9 (57.7 - 96.1) | 73 | 23.1 (3.9 - 42.3) | 168 | 69.0 (61.1 - 77.0) | 81 | 31.0 (23.0 - 38.9) | 222 | 70.7 (62.1 - 79.3) | 95 | 29.3 (20.7 - 37.9) | 123 | 40.5 (29.4 - 51.6) | 194 | 59.5 (48.4 - 70.6) |  |
| Myagdi | 4 | 8.7 (0.0 - 18.7) | 52 | 91.3 (81.3 - 100.0) | 39 | 66.7 (39.8 - 93.6) | 16 | 33.3 (6.4 - 60.2) | 37 | 63.9 (50.2 - 77.6) | 19 | 36.1 (22.4 - 49.8) | 3 | 6.5 (0.0 - 14.0) | 53 | 93.5 (86.0 - 100.0) |  |
| Parbat | 18 | 21.1 (0.0 - 51.9) | 93 | 78.9 (48.1 - 100.0) | 43 | 49.0 (34.9 - 63.2) | 46 | 51.0 (36.8 - 65.1) | 83 | 74.7 (71.9 - 77.5) | 29 | 25.3 (22.5 - 28.1) | 13 | 14.7 (0.0 - 33.7) | 99 | 85.3 (66.3 - 100.0) |  |
| Baglung | 7 | 4.8 (1.0 - 8.5) | 137 | 95.2 (91.5 - 99.0) | 61 | 45.9 (30.7 - 61.2) | 71 | 54.1 (38.8 - 69.3) | 83 | 57.1 (48.6 - 65.5) | 61 | 42.9 (34.5 - 51.4) | 3 | 2.0 (0.0 - 4.4) | 141 | 98.0 (95.6 - 100.0) |  |
| **Province 5** | | | | | | | | | | | | | | | | |  |
| Gulmi | 34 | 22.2 (0.0 - 50.1) | 112 | 77.8 (49.9 - 100.0) | 99 | 75.1 (63.6 - 86.6) | 33 | 24.9 (13.4 - 36.4) | 113 | 77.1 (70.7 - 83.5) | 33 | 22.9 (16.5 - 29.3) | 20 | 13.1 (0.0 - 26.9) | 126 | 86.9 (73.1 - 100.0) |  |
| Palpa | 32 | 36.0 (5.3 - 66.7) | 52 | 64.0 (33.3 - 94.7) | 34 | 59.4 (53.5 - 65.3) | 23 | 40.6 (34.7 - 46.5) | 44 | 49.4 (40.1 - 58.7) | 44 | 50.6 (41.3 - 59.9) | 17 | 18.4 (4.3 - 32.5) | 71 | 81.6 (67.5 - 95.7) |  |
| Nawalparasi^1^ | 113 | 43.8 (25.0 - 62.6) | 182 | 56.2 (37.4 - 75.0) | 121 | 64.5 (49.2 - 79.7) | 72 | 35.5 (20.3 - 50.8) | 200 | 70.9 (59.2 - 82.5) | 95 | 29.1 (17.5 - 40.8) | 76 | 29.7 (16.8 - 42.6) | 219 | 70.3 (57.4 - 83.2) |  |
| Rupandehi | 199 | 59.3 (35.2 - 83.5) | 122 | 40.7 (16.5 - 64.8) | 108 | 45.2 (27.5 - 62.9) | 120 | 54.8 (37.1 - 72.5) | 237 | 73.1 (67.4 - 78.9) | 84 | 26.9 (21.1 - 32.6) | 111 | 32.9 (16.6 - 49.3) | 210 | 67.1 (50.7 - 83.4) |  |
| Kapilbastu | 47 | 25.8 (2.4 - 49.3) | 130 | 74.2 (50.7 - 97.6) | 74 | 55.0 (33.1 - 76.9) | 61 | 45.0 (23.1 - 66.9) | 124 | 70.4 (60.8 - 80.1) | 53 | 29.6 (19.9 - 39.2) | 34 | 18.6 (0.6 - 36.6) | 143 | 81.4 (63.4 - 99.4) |  |
| Arghakhanchi | 1 | 1.2 (0.0 - 3.2) | 83 | 98.8 (96.8 - 100.0) | 38 | 50.0 (36.5 - 63.4) | 37 | 50.0 (36.6 - 63.5) | 50 | 59.4 (53.3 - 65.5) | 34 | 40.6 (34.5 - 46.7) | 0 | 0 | 84 | 100.0 (100.0 - 100.0) |  |
| Pyuthan | 10 | 11.1 (0.7 - 21.4) | 79 | 88.9 (78.6 - 99.3) | 55 | 71.3 (62.1 - 80.6) | 23 | 28.7 (19.4 - 37.9) | 53 | 60.4 (49.7 - 71.1) | 36 | 39.6 (28.9 - 50.3) | 7 | 7.7 (0.0 - 18.1) | 82 | 92.3 (81.9 - 100.0) |  |
| Rolpa | 0 | 0 | 88 | 100.0 (100.0 - 100.0) | 41 | 54.0 (38.3 - 69.7) | 35 | 46.0 (30.3 - 61.7) | 42 | 47.7 (45.8 - 49.6) | 46 | 52.3 (50.4 - 54.2) | 0 | 0 | 88 | 100.0 (100.0 - 100.0) |  |
| Dang | 81 | 43.7 (23.9 - 63.6) | 111 | 56.3 (36.4 - 76.1) | 96 | 67.2 (56.6 - 77.8) | 43 | 32.8 (22.2 - 43.4) | 125 | 64.3 (56.9 - 71.6) | 67 | 35.7 (28.4 - 43.1) | 47 | 25.4 (15.4 - 35.3) | 145 | 74.6 (64.7 - 84.6) |  |
| Banke | 80 | 41.5 (15.1 - 67.9) | 100 | 58.5 (32.1 - 84.9) | 77 | 54.4 (25.8 - 82.9) | 58 | 45.6 (17.1 - 74.2) | 91 | 50.1 (38.9 - 61.4) | 89 | 49.9 (38.6 - 61.1) | 31 | 15.3 (2.3 - 28.2) | 149 | 84.7 (71.8 - 97.7) |  |
| Bardiya | 31 | 22.4 (19.0 - 25.7) | 115 | 77.6 (74.3 - 81.0) | 53 | 64.3 (56.4 - 72.2) | 34 | 35.7 (27.8 - 43.6) | 65 | 44.4 (32.7 - 56.1) | 81 | 55.6 (43.9 - 67.3) | 13 | 9.8 (5.9 - 13.6) | 133 | 90.2 (86.4 - 94.1) |  |
| **Province 6** | | | | | | | | | | | | | | | | |  |
| Rukum^2^ | 32 | 11.8 (0.0 - 27.7) | 139 | 88.2 (72.3 - 100.0) | 115 | 72.8 (61.3 - 84.2) | 49 | 27.2 (15.8 - 38.7) | 39 | 19.3 (6.3 - 32.4) | 132 | 80.7 (67.6 - 93.7) | 9 | 3.2 (0.0 - 7.5) | 162 | 96.8 (92.5 - 100.0) |  |
| Salyan | 23 | 8.6 (0.0 - 19.9) | 207 | 91.4 (80.1 - 100.0) | 87 | 52.6 (34.5 - 70.8) | 75 | 47.4 (29.2 - 65.5) | 134 | 62.2 (51.5 - 72.9) | 96 | 37.8 (27.1 - 48.5) | 15 | 5.6 (0.0 - 13.1) | 215 | 94.4 (86.9 - 100.0) |  |
| Surkhet | 133 | 20.0 (8.5 - 31.4) | 354 | 80.0 (68.6 - 91.5) | 164 | 60.3 (51.8 - 68.9) | 118 | 39.7 (31.1 - 48.2) | 253 | 46.7 (36.2 - 57.1) | 235 | 53.3 (42.9 - 63.8) | 66 | 9.9 (4.0 - 15.7) | 422 | 90.1 (84.3 - 96.0) |  |
| Dailekha | 24 | 7.4 (0.0 - 18.9) | 177 | 92.6 (81.1 - 100.0) | 70 | 37.6 (27.8 - 47.5) | 94 | 62.4 (52.5 - 72.2) | 44 | 21.7 (12.8 - 30.7) | 157 | 78.3 (69.3 - 87.2) | 5 | 1.4 (0.0 - 3.7) | 196 | 98.6 (96.3 - 100.0) |  |
| Jajarkot | 11 | 6.7 (0.0 - 19.4) | 132 | 93.3 (80.6 - 100.0) | 51 | 52.2 (35.9 - 68.6) | 43 | 47.8 (31.4 - 64.1) | 51 | 35.3 (23.1 - 47.6) | 94 | 64.7 (52.4 - 76.9) | 6 | 3.6 (0.0 - 10.3) | 139 | 96.4 (89.7 - 100.0) |  |
| Dolpa | 0 | 0 | 30 | 100.0 (100.0 - 100.0) | 25 | 96.2 (96.2 - 96.2) | 1 | 3.8 (3.8 - 3.8) | 8 | 26.7 (26.7 - 26.7) | 22 | 73.3 (73.3 - 73.3) | 0 | 0 | 30 | 100.0 (100.0 - 100.0) |  |
| Jumla | 16 | 12.4 (0.0 - 36.5) | 65 | 87.6 (63.5 - 100.0) | 36 | 40.8 (22.3 - 59.3) | 45 | 59.2 (40.7 - 77.7) | 24 | 25.8 (13.2 - 38.4) | 57 | 74.2 (61.6 - 86.8) | 8 | 6.2 (0.0 - 18.2) | 73 | 93.8 (81.8 - 100.0) |  |
| Kalikot | 0 | 0 | 57 | 100.0 (100.0 - 100.0) | 25 | 50.9 (46.5 - 55.2) | 24 | 49.1 (44.8 - 53.5) | 10 | 17.4 (13.7 - 21.1) | 47 | 82.6 (78.9 - 86.3) | 0 | 0 | 57 | 100.0 (100.0 - 100.0) |  |
| Mugu | 1 | 1.3 (0.0 - 3.8) | 54 | 98.7 (96.2 - 100.0) | 31 | 64.7 (51.6 - 77.8) | 19 | 35.3 (22.2 - 48.4) | 5 | 6.5 (0.0 - 18.9) | 50 | 93.5 (81.1 - 100.0) | 1 | 1.3 (0.0 - 3.8) | 54 | 98.7 (96.2 - 100.0) |  |
| Humla | 0 | 0 | 30 | 100.0 (100.0 - 100.0) | 26 | 86.7 (86.7 - 86.7) | 4 | 13.3 (13.3 - 13.3) | 4 | 13.3 (13.3 - 13.3) | 26 | 86.7 (86.7 - 86.7) | 0 | 0 | 30 | 100.0 (100.0 - 100.0) |  |
| **Province 7** | | | | | | | | | | | | | | | | |  |
| Bajura | 0 | 0 | 84 | 100.0 (100.0 - 100.0) | 28 | 37.4 (18.4 - 56.5) | 50 | 62.6 (43.5 - 81.6) | 18 | 21.3 (13.3 - 29.4) | 66 | 78.7 (70.6 - 86.7) | 0 | 0 | 84 | 100.0 (100.0 - 100.0) |  |
| Bajhang | 4 | 4.7 (1.1 - 8.3) | 84 | 95.3 (91.7 - 98.9) | 53 | 66.8 (43.6 - 90.0) | 28 | 33.2 (10.0 - 56.4) | 43 | 49.1 (44.6 - 53.5) | 45 | 50.9 (46.5 - 55.4) | 0 | 0 | 88 | 100.0 (100.0 - 100.0) |  |
| Achham | 1 | 0.9 (0.0 - 2.5) | 111 | 99.1 (97.5 - 100.0) | 40 | 38.3 (18.1 - 58.5) | 64 | 61.7 (41.5 - 81.9) | 39 | 35.6 (19.8 - 51.4) | 73 | 64.4 (48.6 - 80.2) | 1 | 0.9 (0.0 - 2.5) | 111 | 99.1 (97.5 - 100.0) |  |
| Doti | 6 | 4.0 (0.7 - 7.3) | 128 | 96.0 (92.7 - 99.3) | 56 | 59.9 (41.9 - 77.8) | 37 | 40.1 (22.2 - 58.1) | 49 | 34.6 (22.7 - 46.6) | 85 | 65.4 (53.4 - 77.3) | 2 | 1.3 (0.0 - 2.7) | 132 | 98.7 (97.3 - 100.0) |  |
| Kailali | 136 | 26.1 (14.6 - 37.7) | 325 | 73.9 (62.3 - 85.4) | 142 | 67.3 (58.7 - 75.9) | 83 | 32.7 (24.1 - 41.3) | 197 | 41.2 (33.4 - 49.0) | 264 | 58.8 (51.0 - 66.6) | 54 | 10.6 (5.1 - 16.2) | 407 | 89.4 (83.8 - 94.9) |  |
| Kanchanpur | 92 | 37.8 (17.4 - 58.2) | 190 | 62.2 (41.8 - 82.6) | 90 | 65.7 (59.7 - 71.7) | 43 | 34.3 (28.3 - 40.3) | 147 | 56.1 (38.5 - 73.6) | 137 | 43.9 (26.4 - 61.5) | 49 | 23.1 (5.0 - 41.2) | 235 | 76.9 (58.8 - 95.0) |  |
| Dadeldhura | 0 | 0 | 57 | 100.0 (100.0 - 100.0) | 24 | 52.9 (36.3 - 69.5) | 21 | 47.1 (30.5 - 63.7) | 23 | 39.3 (0.0 - 79.8) | 34 | 60.7 (20.2 - 100.0) | 0 | 0 | 57 | 100.0 (100.0 - 100.0) |  |
| Baitadi | 4 | 2.1 (0.0 - 5.9) | 144 | 97.9 (94.1 - 100.0) | 63 | 50.5 (35.3 - 65.6) | 58 | 49.5 (34.4 - 64.7) | 76 | 50.9 (44.1 - 57.7) | 72 | 49.1 (42.3 - 55.9) | 3 | 1.5 (0.0 - 4.4) | 145 | 98.5 (95.6 - 100.0) |  |
| Darchula | 27 | 23.6 (0.0 - 63.7) | 88 | 76.4 (36.3 - 100.0) | 59 | 69.0 (57.1 - 80.8) | 26 | 31.0 (19.2 - 42.9) | 55 | 47.8 (45.8 - 49.7) | 60 | 52.2 (50.3 - 54.2) | 10 | 8.7 (0.0 - 23.6) | 105 | 91.3 (76.4 - 100.0) |  |

Note: the percent represents row percent and associated 95% confidence intervals.

^1^Nawalparasi was split into two districts (Nawalpur District in Province No. 4 and Parasi District in Province No. 5)

2 Rukum was split into two districts (Eastern Rukum District in Province No. 5 and Western Rukum District in Province No. 6)
